# Critical Modifying Effect of APOE 4 Allele and Sex on the Prospective Associations between Lipid Species and 10‐year Cognitive Decline in Biracial Community‐dwelling Older Adults: the Chicago Health and Aging Project (CHAP)

**DOI:** 10.1002/alz.089732

**Published:** 2025-01-09

**Authors:** Ted K.S. Ng, Todd Beck, Xiaoran Liu, Pankaja Desai, Thomas Monroe Holland, Klodian Dhana, Kristin R Krueger, Robert S. Wilson, Denis A Evans, Kumar B Rajan

**Affiliations:** ^1^ Rush Institute for Healthy Aging, Chicago, IL USA; ^2^ Rush Alzheimer's Disease Center, Rush University Medical Center, Chicago, IL USA

## Abstract

**Background:**

There have been contradictory findings on the associations between lipids and cognitive decline, which could have been attributed to heterogeneity in APOE allele and sex.

**Method:**

We analyzed a subset of the Chicago Health and Aging Project, comprising 3,496 biracial community‐dwelling older adults (58% African American & 64% women; mean follow‐up=4.6 years) with lipid panel and longitudinal cognitive tests. We ran adjusted mixed‐effects models and examined APOE allelic and sex differences.

**Result:**

Total cholesterol and triglycerides, independently, was associated with longitudinal cognitive decline only in ε4 carriers. The associations of total cholesterol and, independently, triglycerides with longitudinal cognitive decline was driven by men and women ε4 carriers, respectively. LDL was associated with cognitive decline only in men ε4 carriers.

**Conclusion:**

The associations of lipids and cognitive decline were only present in ε4 carriers and are further dependent on and diverged according to sex, highlighting their importance in risk prediction and treatment. Hence, apart from examining lipid panel, it is critical to simultaneously consider APOE 4 status and sex to better inform precision medicine, particularly risk prediction and treatment.

